# Up-Converting K_2_Gd(PO_4_)(WO_4_):20%Yb^3+^,Ho^3+^ Phosphors for Temperature Sensing

**DOI:** 10.3390/ma16030917

**Published:** 2023-01-18

**Authors:** Julija Grigorjevaite, Arturas Katelnikovas

**Affiliations:** Institute of Chemistry, Faculty of Chemistry and Geosciences, Vilnius University, Naugarduko 24, LT-03225 Vilnius, Lithuania

**Keywords:** luminescence, temperature-dependent up-conversion emission, energy transfer, CIE1931 color coordinates

## Abstract

Inorganic luminescent materials that can be excited with NIR radiation and emit in the visible spectrum have recently gained much scientific interest. Such materials can be utilized as anti-counterfeiting pigments, luminescent thermometers, bio-imaging agents, etc. In this work, we report the synthesis and optical properties of K_2_Gd(PO_4_)(WO_4_):Ho^3+^ and K_2_Gd(PO_4_)(WO_4_):20%Yb^3+^,Ho^3+^ powders. The single-phase samples were prepared by the solid-state reaction method, and the Ho^3+^ concentration was changed from 0.5% to 10% with respect to Gd^3+^. It is interesting to note that under 450 nm excitation, no concentration quenching was observed in K_2_Gd(PO_4_)(WO_4_):Ho^3+^ (at least up to 10% Ho^3+^) samples. However, adding 20% Yb^3+^ has caused a gradual decrease in Ho^3+^ emission intensity with an increase in its concentration. It turned out that this phenomenon is caused by the increasing probability of Ho^3+^ → Yb^3+^ energy transfer when Ho^3+^ content increases. K_2_Gd(PO_4_)(WO_4_):20%Yb^3+^,0.5%Ho^3+^ sample showed exceptionally high up-conversion (UC) emission stability in the 77–500 K range. The UC emission intensity reached a maximum at ca. 350 K, and the intensity at 500 K was around four times stronger than the intensity at 77 K. Moreover, the red/green emission ratio gradually increased with increasing temperature, which could be used for temperature sensing purposes.

## 1. Introduction

In recent decades, the up-converting luminescent materials based on lanthanide ions attracted much scientific interest due to their outstanding luminescence properties. The up-converting luminescent materials are widely used in a variety of applications, such as solar energy [1,2], anti-counterfeiting pigments [3], temperature sensors [4], fluorescence probes [5], bio-imaging [6], cancer therapeutics [7], etc. Usually, the inorganic up-converting luminescent materials contain at least two incorporated lanthanide ions: one as a sensitizer, typically Yb^3+^, and another as an emitter, such as Er^3+^ [8,9], Ho^3+^ [10,11,12,13], Tm^3+^ [14,15,16], etc. Another critical step in preparing up-converting phosphors is the selection of an appropriate host matrix. The researchers usually focus on the fluoride (or another halide) and tellurate glass hosts because they possess relatively low phonon frequencies, which, in turn, reduces the energy losses via non-radiative processes and yield high up-conversion luminescence efficiencies [10]. Among the fluoride-based hosts for up-converting phosphors, the (Li,Na,K)(La,Y,Gd,Lu)F_4_ host is probably the most studied [17,18,19]. On the other hand, these host matrices have several drawbacks, for instance, insufficient thermal, physical, and chemical stability. They are also toxic, hygroscopic, and so on [20]. Therefore, other inorganic matrices, such as tungstates, molybdates, vanadates, titanates, etc., are gaining more and more attention [21]. Among these materials, tungstates are widely studied as luminescent materials due to their excellent thermal and chemical stability [22,23,24]. For this reason, the well-known K_2_Gd(PO_4_)(WO_4_) [25,26,27] was chosen as a host matrix in this study.

There is a long list of lanthanide ions used as emitters in up-converting phosphors. We want to stress that Ho^3+^ is one of the most exciting lanthanide ions due to its unique energy level structure. Besides, Ho^3+^ energy levels match well with the energy levels of Yb^3+^, thus, the Yb^3+^/Ho^3+^ couple could be one of the choices for preparing up-converting phosphors [28]. Solely Ho^3+^ doped matrices could be used as a down-conversion (DC) material if Ho^3+^ is directly excited with 450 nm radiation. Furthermore, Yb^3+^/Ho^3+^ co-doped materials are bi-functional since they can be suitable for up-conversion and down-conversion (DC) applications.

In this contribution, we report the successful synthesis of K_2_Gd(PO_4_)(WO_4_) host lattice co-doped with Yb^3+^ and Ho^3+^. Yb^3+^ concentration was fixed at 20% with respect to Gd^3+^, whereas Ho^3+^ concentration was varied between 0.5% and 10%. The influence of Ho^3+^ concentration on luminescence properties was investigated and discussed. The obtained results show that this particular compound could be used as an NIR-excited luminescent security pigment.

## 2. Materials and Methods

A series of K_2_Gd(PO_4_)(WO_4_) samples doped with Ho^3+^ and co-doped with 20% Yb^3+^ and Ho^3+^ (where Ho^3+^ concentration was 0%, 0.5%, 1%, 2%, 5%, and 10% with respect to Gd^3+^) were synthesized by the solid-state reaction method. The starting materials, namely, Gd_2_O_3_ (99.99% Tailorlux, Münster, Germany), K_2_CO_3_ (99+% Acros Organics, Geel, Belgium), NH_4_H_2_PO_4_ (99% Reachem Slovakia, Petržalka, Slovakia), WO_3_ (99+% Acros Organics), Yb_2_O_3_ (99.99% Alfa Aesar, Haverhill, MA, USA), and Ho_2_O_3_ (99.99% Alfa Aesar) were weighed and blended in stoichiometric amounts. The powders were blended in an agate mortar. A few milliliters of acetone were added to accelerate the homogenization. The mixed reagents were poured into the porcelain crucible and annealed at 873 K temperature for 10 h in the air in a muffle furnace. Subsequently, the annealing process was repeated twice more with intermediate grinding of the products.

The structural analysis of the synthesized materials was performed using a Rigaku MiniFlexII diffractometer working on a Bragg–Brentano-focusing geometry (Tokyo, Japan). SEM images were taken on a field-emission scanning electron microscope FE-SEM Hitachi SU-70. The optical properties (room temperature reflection, excitation, and emission spectra; temperature-dependent emission spectra; PL decay) were investigated employing the modular Edinburgh Instruments FLS980 spectrometer. The instrumental parameters for each measurement are summarized in Tables S1–S4.

Rietveld refinement of the XRD patterns was performed using FullProf Suite software (version 2 December 2022). Peak profiles were modeled using a pseudo-Voigt peak shape. A 24-term Chebyshev-type background function was used. Other experimental parameters refined were the instrument zero, scale factor, lattice parameters, preferred orientation, and the peak shape parameters *u*, *v*, *w*, *γ*_0_, and *γ*_1_. For Rietveld fits, the K_2_Ho(PO_4_)(WO_4_) structure (PDF-4+ (ICDD) 04-015-9304) reported by Terebilenko et al. [29] was used and atomic coordinates were refined.

## 3. Results and Discussion

**Figure 1:**
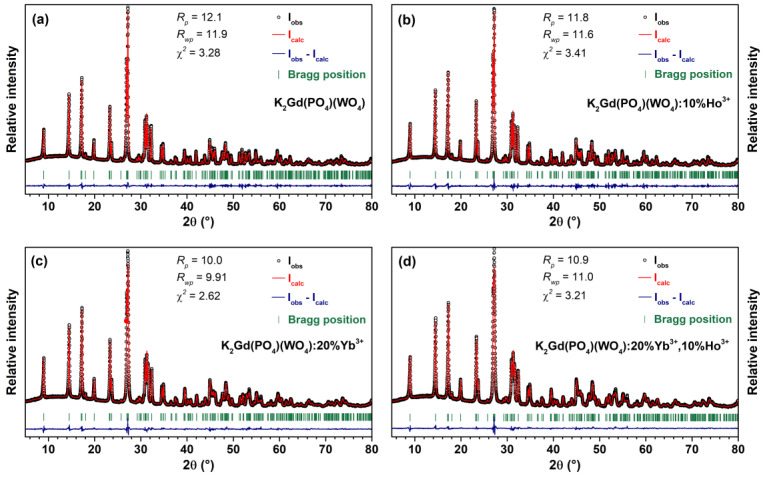
Rietveld refinement of undoped K_2_Gd(PO_4_)(WO_4_) (**a**), K_2_Gd(PO_4_)(WO_4_):10%Ho^3+^ (**b**), K_2_Gd(PO_4_)(WO_4_):20%Yb^3+^ (**c**), and K_2_Gd(PO_4_)(WO_4_):20%Yb^3+^,10%Ho^3+^ (**d**) XRD patterns.

The phase purity of the synthesized K_2_Gd(PO_4_)(WO_4_):x%Ho^3+^ and K_2_Gd(PO_4_)(WO_4_):20%Yb^3+^,x%Ho^3+^ samples was investigated by recording powder XRD patterns. In order to extract the lattice parameters of the synthesized compounds, the Rietveld refinement of the recorded powder XRD patterns was performed. The Rietveld refinement of undoped K_2_Gd(PO_4_)(WO_4_), K_2_Gd(PO_4_)(WO_4_):10%Ho^3+^, K_2_Gd(PO_4_)(WO_4_):20%Yb^3+^, and K_2_Gd(PO_4_)(WO_4_):20%Yb^3+^,10%Ho^3+^ samples are shown in Figure 1. The calculated lattice parameters of K_2_Gd(PO_4_)(WO_4_), K_2_Gd(PO_4_)(WO_4_):10%Ho^3+^, K_2_Gd(PO_4_)(WO_4_):20%Yb^3+^, and K_2_Gd(PO_4_)(WO_4_):20%Yb^3+^,10%Ho^3+^ samples are given in Table S5. The lattice parameters decrease with increasing Yb^3+^ and Ho^3+^ content in the structure which, in fact, was expected since both ions are smaller than Gd^3+^. The XRD patterns of all the synthesized samples were similar and matched exceptionally well with the reference pattern, indicating that single-phase compounds were obtained. The synthesized K_2_Gd(PO_4_)(WO_4_):Yb^3+^,Ho^3+^ compounds crystalize in an orthorhombic crystal structure and adopt the Ibca (#73) space group [30]. The crystal structure of the K_2_Gd(PO_4_)(WO_4_) compound is constructed by PO_4_ and WO_4_ tetrahedrons and K^+^ and Gd^3+^ polyhedrons (both K^+^ and Gd^3+^ are eight-fold coordinated). Since the ionic radii of eight-coordinated Gd^3+^ (r = 1.053 Å), Yb^3+^ (r = 0.985 Å), and Ho^3+^ (r = 1.015 Å) [31] are very similar, we assumed that Yb^3+^ and Ho^3+^ occupied the Gd^3+^ sites in the crystal lattice. It is also worth mentioning that the K_2_Gd(PO_4_)(WO_4_) crystal structure is very versatile from a chemical point of view. For instance, potassium ions in the structure can be easily replaced by sodium [32] and rubidium [33] ions. Gd^3+^, in turn, can be replaced by nearly all rare-earth ions [34] as well as Y^3+^ [35], and Bi^3+^ [36]. WO_4_ groups can be also exchanged by MoO_4_ groups [36] making virtually endless possibilities for modification of chemical structures’ chemical composition.

The morphological features of the synthesized compounds were investigated by taking SEM images. The SEM images of K_2_Gd(PO_4_)(WO_4_):20%Yb^3+^, K_2_Gd(PO_4_)(WO_4_):10%Ho^3+^, and K_2_Gd(PO_4_)(WO_4_):20%Yb^3+^,10%Ho^3+^ specimens are depicted in Figure S1. All three SEM images demonstrate that the synthesized powders consist of aggregated microparticles with irregular shapes. Moreover, the powder particles were virtually identical, and no differences in crystallite size or morphology were observed with varying Yb^3+^ or Ho^3+^ concentrations.

**Figure 2:**
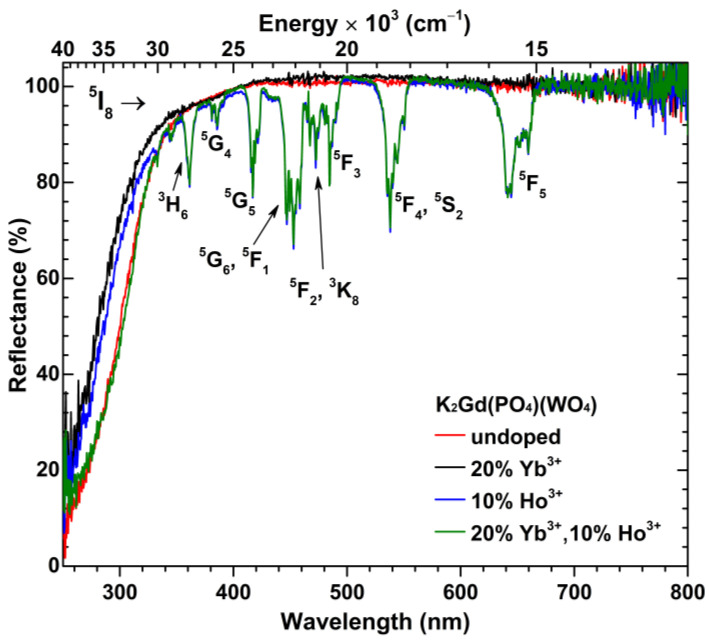
Reflectance spectra of K_2_Gd(PO_4_)(WO_4_) (red line), K_2_Gd(PO_4_)(WO_4_):20%Yb^3+^ (black line), K_2_Gd(PO_4_)(WO_4_):10%Ho^3+^ (blue line), and K_2_Gd(PO_4_)(WO_4_):20%Yb^3+^,10%Ho^3+^ (green line) specimens.

The body color of the undoped K_2_Gd(PO_4_)(WO_4_) and 20% Yb^3+^ doped samples was white, indicating that both compounds do not absorb in the visible spectrum range. The Ho^3+^ doped samples, in turn, possessed a yellowish body color under sunlight and the reddish body color under fluorescent lamp (FL) illumination. The yellowish body color is caused by many strong absorption lines of Ho^3+^ in the visible spectrum, whereas the reddish body color is a result of Ho^3+^ excitation by the fluorescent lamp leading to red emission. The reflection spectra of undoped, 20% Yb^3+^ doped, and 10% Ho^3+^ doped, together with 20% Yb^3+^ and 10%Ho^3+^ co-doped samples are shown in Figure 2. The reflectance spectra were measured in a 250–800 nm range. The reflectance spectra of Ho^3+^ doped samples possess several sets of absorption lines typical to Ho^3+^, i.e., ^5^I_8_ → ^3^H_6_ (ca. 355–368 nm), ^5^I_8_ → ^5^G_4_ (ca. 380–390 nm), ^5^I_8_ → ^5^G_5_ (ca. 410–426 nm), ^5^I_8_ → ^5^G_6_;^5^F_1_ (ca. 440–464 nm), ^5^I_8_ → ^5^F_2_;^3^K_8_ (ca. 466–480 nm), ^5^I_8_ → ^5^F_3_ (ca. 483 nm), ^5^I_8_ → ^5^F_4_;^5^S_2_ (ca. 525–555 nm), and ^5^I_8_ → ^5^F_5_ (ca. 624–665 nm). The broad absorption band in the UV range (around 300 nm) can be assigned to the O^2−^ → W^6+^ charge transfer transition in the host lattice [37].

**Figure 3:**
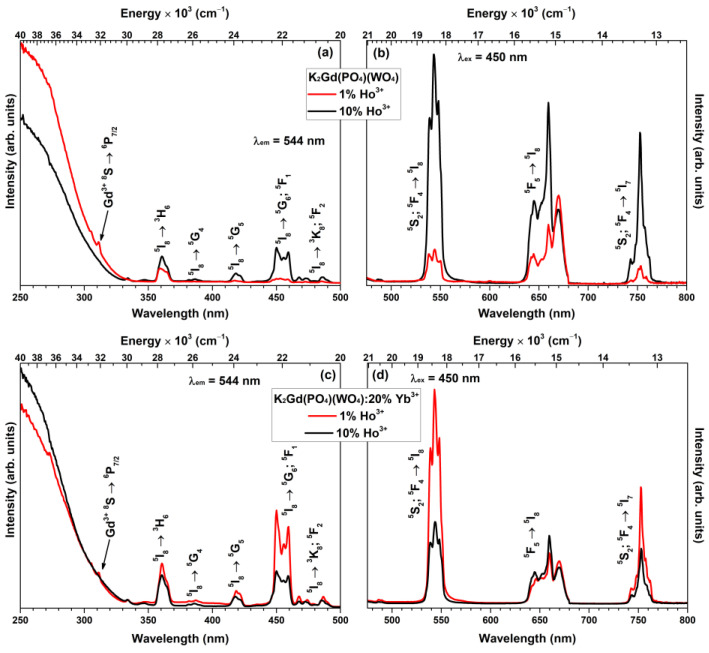
(**a**) Excitation (λ_em_ = 544 nm) and (**b**) emission (λ_ex_ = 450 nm) spectra of K_2_Gd(PO_4_)(WO_4_) doped with 1% and 10% Ho^3+^. (**c**) Excitation (λ_em_ = 544 nm) and (**d**) emission (λ_ex_ = 450 nm) spectra of K_2_Gd(PO_4_)(WO_4_):20%Yb^3+^ doped with 1% and 10% Ho^3+^.

The excitation (λ_em_ = 544 nm) spectra of K_2_Gd(PO_4_)(WO_4_):Ho^3+^ and K_2_Gd(PO_4_)(WO_4_):20%Yb^3+^,Ho^3+^ (where the Ho^3+^ concentration is 1% and 10%) samples were measured in the 250–500 nm range and are depicted in Figure 3a,c, respectively. The measured spectra consist of several sets of excitation lines which originated from the typical Ho^3+^ ground state (^5^I_8_) transitions to ^3^H_6_ (ca. 355–368 nm), ^5^G_4_ (ca. 380–390 nm), ^5^G_5_ (ca. 410–426 nm), ^5^G_6_ + ^5^F_1_ (ca. 440–464 nm), and ^3^K_8_ + ^5^F_2_ (ca. 466–480 nm) [38,39]. The highest excitation line intensity for solely Ho^3+^ doped samples was observed for the 10% doped sample. Moreover, a weak excitation line attributed to the ^8^S → ^6^P_7/2_ (ca. 311 nm) transition of Gd^3+^ [40] is also visible in the excitation spectra. This indicates that some Gd^3+^ → Ho^3+^ energy transfer occurs in the given host matrix. The same Gd^3+^ line was also observed in the K_2_Gd(PO_4_)(WO_4_):20%Yb^3+^,Ho^3+^ excitation spectra. Besides, the strongest Ho^3+^ excitation line intensity for Yb^3+^-containing samples was observed when the Ho^3+^ concentration was fixed at 1%. The broad excitation band in the range of 250–330 nm is attributed to the O^2–^ to W^6+^ charge-transfer transition within the WO_4_^2−^. Such transitions are typical for the tungstate compounds in this spectral range and reported by many authors [41,42].

The emission spectra (λ_ex_ = 450 nm) of K_2_Gd(PO_4_)(WO_4_) and K_2_Gd(PO_4_)(WO_4_):20%Yb^3+^ samples doped with 1% and 10% Ho^3+^ were measured in 450–800 nm range and are presented in Figure 3b,d, respectively. Three sets of emission lines were observed in the emission spectra and the lines are attributed to Ho^3+^ transitions: ^5^S_2_ + ^5^F_4_ → ^5^I_8_ (ca. 530–555 nm), ^5^F_5_ → ^5^I_8_ (ca. 630–670 nm), and ^5^S_2_ + ^5^F_4_ → ^5^I_7_ (ca. 740–765 nm). Among solely Ho^3+^ doped samples, the one doped with 10% showed the most intensive emission. This finding is rather surprising since Ho^3+^ energy levels are exceptionally suitable for cross-relaxation processes [43,44]. However, the opposite tendency was observed when Yb^3+^ was incorporated into the host matrix. The sample doped with 1% Ho^3+^ showed the most intensive emission in this case. This is related to the increasing Ho^3+^ → Yb^3+^ energy transfer probability at higher Ho^3+^ concentrations resulting in a decrease in Ho^3+^ emission intensity. Similar results were also obtained by other authors working with other host matrices [10].

**Figure 4:**
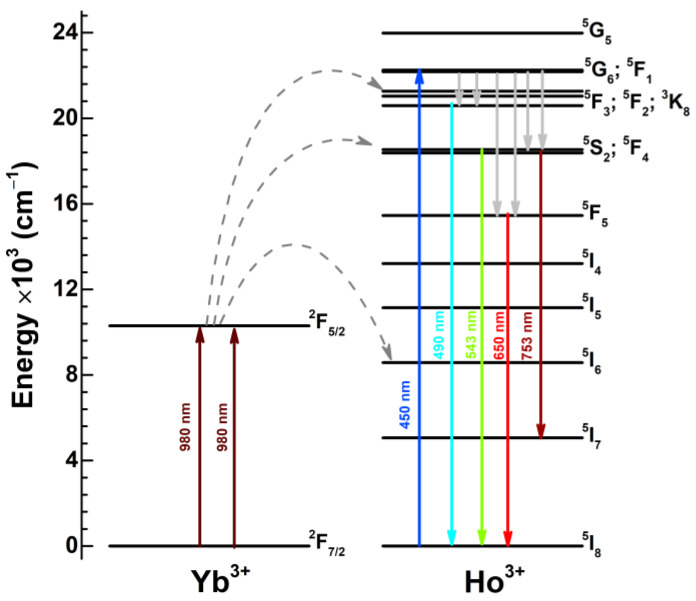
Schematic energy level diagram of energy transitions in Yb^3+^ and Ho^3+^.

The schematic energy level diagram with the main Yb^3+^ and Ho^3+^ transitions is depicted in Figure 4. Samples containing Ho^3+^ could be directly excited to ^5^G_6_; ^5^F_1_ energy levels with the 450 nm excitation radiation (blue arrow). After relaxation to the lower-lying energy levels, emission in the cyan, green, red, and deep red spectral regions occurs. If the Ho^3+^ doped sample also contains Yb^3+^, the sample can be excited with the NIR radiation via the Yb^3+^ → Ho^3+^ energy transfer.

Yb^3+^ transfers energy to Ho^3+^ through several steps. First of all, the Yb^3+^ ion absorbs one NIR photon and transfers it to the ^5^I_6_ level of Ho^3+^ (I step). The subsequent NIR photon from Yb^3+^ excites the electrons within the ^5^I_6_ level to the ^5^S_2_ and ^5^F_4_ levels of Ho^3+^ (II step). of These transitions can be written. Since the UC emission spectra also contain emission lines from the ^5^F_2;3_ level, indicating that this level is also slightly populated through the cooperative sensitization as was also reported by the other researchers (III step) [45]. The mentioned UC mechanism can be expressed through these transitions [45,46,47]:

I step   ^2^F_5/2_(Yb^3+^) + ^5^I_8_(Ho^3+^) → ^2^F_7/2_(Yb^3+^) + ^5^I_6_(Ho^3+^)
II step  ^2^F_5/2_(Yb^3+^) + ^5^I_6_(Ho^3+^) → ^2^F_7/2_(Yb^3+^) + ^5^S_2_;^5^F_4_(Ho^3+^)
III step ^2^F_5/2_(Yb^3+^) + ^5^I_8_(Ho^3+^) → ^2^F_7/2_(Yb^3+^) + ^5^F_3_(Ho^3+^)

After the population of the mentioned energy levels of Ho^3+^, the emission from these levels occurs in the green, red, and deep-red spectral areas.

**Figure 5:**
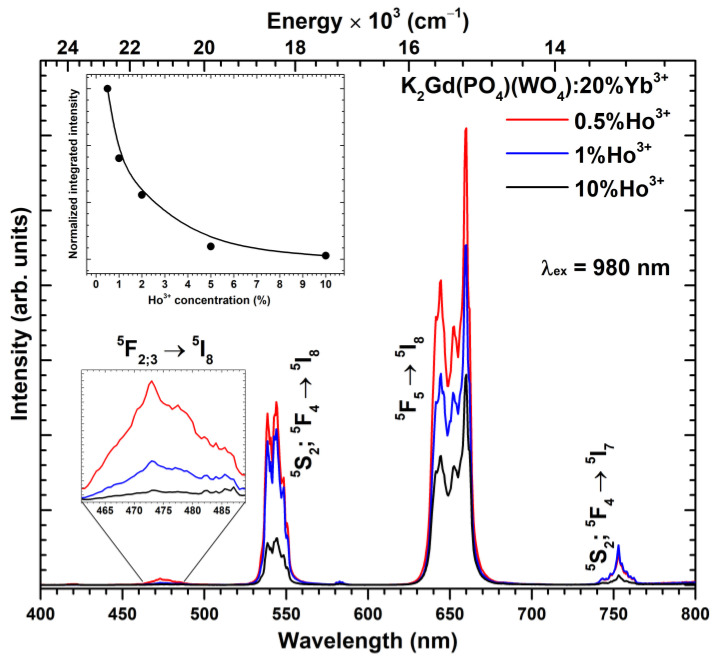
Up-conversion emission spectra of K_2_Gd(PO_4_)(WO_4_) as a function of Ho^3+^ concentration, under the 980 nm excitation. The inset graph shows Ho^3+^ concentration-dependent normalized integrated emission.

Up-conversion emission spectra (λ_ex_ = 980 nm) of K_2_Gd(PO_4_)(WO_4_):20%Yb^3+^,Ho^3+^ and normalized integrated emissions as a function of Ho^3+^ concentration are depicted in Figure 5. As shown in Figure 1, Ho^3+^ does not have energy levels that could be directly excited with the 980 nm laser radiation; therefore, in this case, Yb^3+^ absorbs the laser radiation and transfers the energy to Ho^3+^. The measured Ho^3+^ up-conversion spectra are very similar to those when Ho^3+^ was directly excited with blue radiation (please refer to Figure 4). Typical Ho^3+^ emission lines were observed in up-conversion spectra measured in the 400–800 nm range, i.e., ^5^F_2;3_ → ^5^I_8_ (ca. 460–488 nm), ^5^S_2_;^5^F_4_ → ^5^I_8_ (ca. 530–555 nm), ^5^F_5_ → ^5^I_8_ (ca. 630–670 nm), and ^5^S_2_;^5^F_4_ → ^5^I_7_ (ca. 740–765 nm). The most intensive emission lines were observed for the ^5^F_5_ → ^5^I_8_ transition in the red spectral region. The highest up-conversion emission intensity was observed for the sample doped with 0.5% Ho^3+^. The up-conversion emission intensity decreases with further increasing the Ho^3+^ concentration. The normalized integrated emission of the samples (please refer to the inset in Figure 5) confirms that the total up-conversion emission intensity drastically decreases with increasing Ho^3+^ concentrations. The up-conversion emission intensity decrease with increasing Ho^3+^ concentration is caused by the increasing probability of Ho^3+^ → Yb^3+^ energy transfer, as discussed above. The digital images of K_2_Gd(PO_4_)(WO_4_):20%Yb^3+^,Ho^3+^ up-conversion luminescence (λ_ex_ = 980 nm laser) as a function of Ho^3+^ concentration are given in Figure S2.

**Figure 6:**
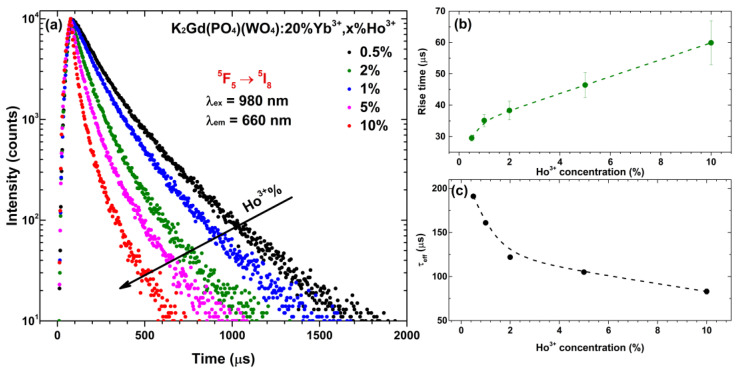
(**a**) Up-conversion PL decay curves (λ_ex_ = 980 nm, λ_em_ = 660 nm) of K_2_Gd(PO_4_)(WO_4_):20%Yb^3+^,Ho^3+^ as a function of Ho^3+^ concentration, (**b**) PL rise time and (**c**) effective decay lifetime (*τ_eff_*) values of the same samples as a function of Ho^3+^ concentration.

In order to better understand the up-conversion process of the prepared materials, the PL decay curves (λ_ex_ = 980 nm, λ_em_ = 660 nm) of the most intensive emission peak (^5^F_5_ → ^5^I_8_ transition) were measured. The recorded UC PL decay curves of K_2_Gd(PO_4_)(WO_4_):20%Yb^3+^,Ho^3+^ samples as a function of Ho^3+^ concentration are shown in Figure 6a–c, in turn, show the calculated PL rise time and *τ_eff_* values of the same samples, respectively. The PL decay curves become steeper with increasing Ho^3+^ concentration, indicating that PL lifetime values decrease. This indeed is true since the calculated *τ_eff_* values decreased from 191 μs for 0.5% Ho^3+^ doped samples to 83 μs for 10% Ho^3+^ doped samples (please refer to Figure 6c). The opposite tendency, however, was observed for the PL rise time values, which increased with increasing Ho^3+^ concentration. The exact calculated PL rise time and lifetime values, together with standard deviations, are summarized in Table S6. The calculated UC PL lifetime values for ^5^F_5_ → ^5^I_8_ transition are very similar to the ones reported by other authors in a wide variety of materials, i.e., oxides, phosphates, titanates, silicates, tungsten tellurite glasses, and even fluorides. For instance, Guo et al. reported that the UC PL lifetime value of BaGdF_5_:20%Yb^3+^,1%Ho^3+^ is around 131 μs [28] which is close to the 122 μs for our K_2_Gd(PO_4_)(WO_4_):20%Yb^3+^,2%Ho^3+^ sample. Rather similar UC PL lifetimes were also observed in Sr_3_Y(PO_4_)_3_:10%Yb^3+^,2%Ho^3+^ (261 μs) and BaTiO_3_:3%Yb^3+^,0.2%Ho^3+^ (155 μs for cubic phase and 125 μs for tetragonal phase) compounds which were reported by Liu et al. [48] and Mahata et al. [11], respectively. However, there are also host matrixes where Ho^3+^ UC PL lifetimes are much shorter, for instance, La_9.31_(Si_1.04_O_4_)_6_O_2_:20%Yb^3+^,1%Ho^3+^ (around 18 μs) [49] and tungsten–tellurite glass (around 33 μs) [50]. An overview of these materials is given in Table 1.

**Figure 7:**
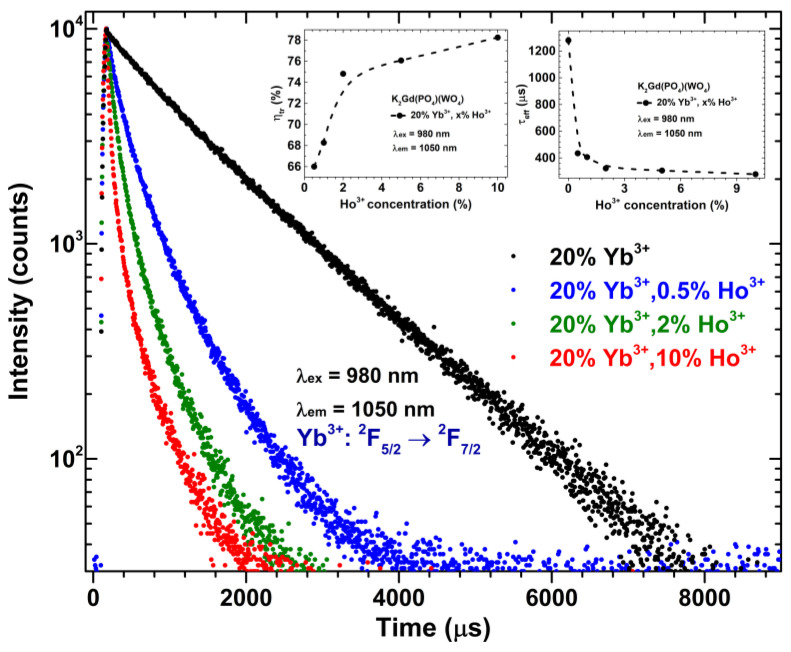
Yb^3+^ decay curves of K_2_Gd(PO_4_)(WO_4_):20%Yb^3+^ as a function of Ho^3+^ concentration. Inset graphs show the energy transfer efficiency (*η_tr_*) and *τ_eff_* values as a function of Ho^3+^ concentration.

In order to evaluate the PL lifetime values of Yb^3+^ in the prepared samples, the PL curves under the 980 nm laser excitation were recorded by monitoring emission at 1050 nm. The obtained PL decay curves are depicted in Figure 7. The PL lifetime values of Yb^3+ 2^F_5/2_ → ^2^F_7/2_ transition drastically decrease from 1279 ± 23 μs to 278 ± 6 μs with increasing Ho^3+^ concentration from 0% to 10%; the tendency and exact values are represented in Figure 7 inset and Table S7. Such an abrupt decrease of Yb^3+^ PL lifetime with increasing Ho^3+^ concentration is related to the Yb^3+^ → Ho^3+^ energy transfer. The energy transfer efficiency (*η_tr_*) was calculated from the Yb^3+^ PL lifetime values by the following formula [51]:

(1) $$\eta_{tr}=\left(1-\frac{\tau_{Yb-Ho}}{\tau_{Yb}}\right)\times 100\%$$

where *τ_Yb−Ho_* and *τ_Yb_* are Yb^3+^ PL lifetime values of ^2^F_5/2_ → ^2^F_7/2_ transition in the presence and absence of Ho^3+^, respectively. The calculated *η_tr_* values are summarized in Figure 7 inset and Table S7. The *η_tr_* increases from 66% to 78% when changing Ho^3+^ concentration from 0.5% to 10%, respectively. The obtained results show that the energy transfer from Yb^3+^ to Ho^3+^ is very efficient in this particular host matrix.

**Figure 8:**
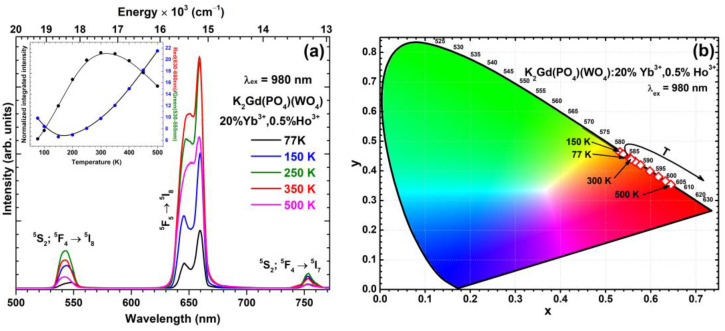
(**a**) Temperature-dependent up-conversion emission spectra of K_2_Gd(PO_4_)(WO_4_):20%Yb^3+^,0.5%Ho^3+^ sample under the 980 nm laser excitation. The inset shows normalized integrated emission intensity and the ratio between Red (630–680 nm) and Green (530–555 nm) emission integrals (lines were drawn to guide the eye). (**b**) temperature-dependent color coordinates of 0.5% Ho^3+^ doped sample.

The temperature-dependent up-conversion emission spectra were recorded in the 77–500 K temperature range to evaluate samples’ performance at high temperatures. The temperature-dependent up-conversion emission (λ_ex_ = 980 nm) spectra of K_2_Gd(PO_4_)(WO_4_):20%Yb^3+^,0.5%Ho^3+^ sample along with normalized integrated emission and red/green emission ratio are presented in Figure 8a. Ho^3+^ emission increases with increasing temperature from 77 to 350 K and then decreases with further temperature increases. However, it should be noted that the integrated UC emission intensity at 500 K is around four times higher than the emission at 77 K. The normalized integrated UC emission intensity reaches a maximum of around 300 K and then decreases. The red/green emission ratio decreases in the temperature range from 77 to 150 K and increases with further temperature increase. This shows that the green emission is quenched faster with increasing temperature compared to the red emission. Such temperature-dependent UC emission spectra feature could be used for luminescent temperature sensing. Moreover, the red/green ratio change is also reflected by a color change from orange-red (at 77 K) to orange (at 300 K) and then to a deep-red region with a further temperature increase to 500 K. The color point shifting could be observed in the CIE1931 color space diagram. All the calculated color points are near the edge of the CIE1931 color space diagram, showing the high color purity of the prepared samples. The exact calculated color coordinates of the prepared samples are tabulated in Table S8.

## 4. Conclusions

The single-phase K_2_Gd(PO_4_)(WO_4_):Ho^3+^ and K_2_Gd(PO_4_)(WO_4_):20%Yb^3+^,Ho^3+^ powders, where Ho^3+^ concentration varied from 0.5% to 10%, were successfully prepared by the solid-state reaction method. Solely Ho^3+^ doped samples under 450 nm excitation showed no concentration quenching (at least up to 10% Ho^3+^). However, adding 20% Yb^3+^ caused a gradual decrease in Ho^3+^ emission (under 450 nm excitation) intensity with an increase in its concentration. It turned out that this phenomenon is caused by the increasing probability of Ho^3+^ → Yb^3+^ energy transfer when Ho^3+^ content increases. This was also confirmed by the fact that the strongest UC emission was observed for the K_2_Gd(PO_4_)(WO_4_):20%Yb^3+^,0.5%Ho^3+^ sample. Moreover, the K_2_Gd(PO_4_)(WO_4_):20%Yb^3+^, 0.5%Ho^3+^ sample showed exceptionally high up-conversion (UC) emission stability in the 77–500 K range. The UC emission intensity reached a maximum at ca. 350 K, and the intensity at 500 K was around four times stronger compared to the intensity at 77 K. Furthermore, the red/green emission ratio gradually increased with increasing temperature from 150 to 500 K, and it could be used for temperature sensing purposes. This also indicates that green emission is quenched faster than red emission in Ho^3+^ temperature-dependent UC emission spectra. The bright UC emission of the synthesized phosphors could also be employed in preparing anti-counterfeiting pigments.

## Figures and Tables

**Table 1:** UC PL lifetime values of inorganic host matrixes doped with Ho^3+^.

Upconverting Material	UC PL Lifetime (μs)	Ref.
BaGdF_5_:20%Yb^3+^,1%Ho^3+^	131	[28]
Sr_3_Y(PO_4_)_3_:10%Yb^3+^,2%Ho^3+^	261	[48]
cubic BaTiO_3_:3%Yb^3+^,0.2%Ho^3+^	155	[11]
tetragonal BaTiO_3_:3%Yb^3+^,0.2%Ho^3+^	125	[11]
La_9.31_(Si_1.04_O_4_)_6_O_2_:20%Yb^3+^,1%Ho^3+^	18	[49]
TeO_2_–WO_3_ glass	33	[50]
K_2_Gd(PO_4_)(WO_4_):20%Yb^3+^,0.5%Ho^3+^	191	This work
K_2_Gd(PO_4_)(WO_4_):20%Yb^3+^,10%Ho^3+^	83	This work

## Data Availability

The data presented in this study are available on request from the corresponding author.

## Supplementary Materials

The following supporting information can be downloaded at: https://www.mdpi.com/article/10.3390/ma16030917/s1, Table S1. Spectrometer settings for measuring reflection spectra of K_2_Gd(PO_4_)(WO_4_):20%Yb^3+^,x%Ho^3+^ phosphors. Table S2. Spectrometer settings for measuring excitation spectra of K_2_Gd(PO_4_)(WO_4_):20%Yb^3+^,x%Ho^3+^ phosphors. Table S3. Spectrometer settings for measuring emission spectra of K_2_Gd(PO_4_)(WO_4_):20%Yb^3+^,x%Ho^3+^ phosphors. Table S4. Spectrometer settings for measuring up-conversion emission spectra of K_2_Gd(PO_4_)(WO_4_):20%Yb^3+^,x%Ho^3+^ phosphors. Table S5. Lattice parameters of K_2_Gd(PO_4_)(WO_4_), K_2_Gd(PO_4_)(WO_4_):10%Ho^3+^, K_2_Gd(PO_4_)(WO_4_):20%Yb^3+^, and K_2_Gd(PO_4_)(WO_4_):20%Yb^3+^,10%Ho^3+^ samples derived from Rietveld refinement analysis. Table S6. Effective up-conversion PL rise time and lifetime values of K_2_Gd(PO_4_)(WO_4_):20%Yb^3+^ phosphors as a function of Ho^3+^ concentration (λ_ex_ = 980 nm, λ_em_ = 660 nm). Table S7. Effective up-conversion PL decay lifetime values and energy transfer efficiency (*η_tr_*) of K_2_Gd(PO_4_)(WO_4_):20%Yb^3+^,x%Ho^3+^ phosphors as a function of Ho^3+^ concentration (λ_ex_ = 980 nm, λ_em_ = 1050 nm). Table S8. Color coordinates (CIE 1931 color space) of K_2_Gd(PO_4_)(WO_4_):20%Yb^3+^,0.5%Ho^3+^ as a function of temperature (λ_ex_ = 980 nm). Figure S1. SEM images of K_2_Gd(PO_4_)(WO_4_):20%Yb^3+^ (a), K_2_Gd(PO_4_)(WO_4_):10%Ho^3+^ (b), and K_2_Gd(PO_4_)(WO_4_):20%Yb^3+^,10%Ho^3+^ (c), Figure S2. Digital images of the K_2_Gd(PO_4_)(WO_4_):20%Yb^3+^,Ho^3+^ up-conversion luminescence (λ_ex_ = 980 nm laser) as a function of Ho^3+^ concentration.
