# Supplementary material for: Up-Converting K2Gd(PO4)(WO4):20%Yb3+,Ho3+ Phosphors for Temperature Sensing
Source: Materials (Basel). 2023 Jan 18;16(3):917. doi: 10.3390/ma16030917 (PMC9917978; doi:10.3390/ma16030917)
Supplement: Supplementary file 1 [file materials-16-00917-s001.zip › materials-2080053-supplementary.pdf]

materials

Supporting information

Up-Converting $K_2Gd(PO_4)(WO_4)$:20%Yb^{3+},Ho^{3+} Phosphors for Temperature Sensing

Julija Grigorjevaite and Arturas Katelnikovas *

Table S1. Spectrometer settings for measuring reflectance spectra of $K_2Gd(PO_4)(WO_4)$:20%Yb^{3+},x%Ho^{3+} phosphors.

**$K_2Gd(PO_4)(WO_4)$:20%Yb^{3+},x%Ho^{3+}**	
**Parameters**	
EmBW	0.15 nm
ExBW	4.00 nm
Step	0.50 nm
Integration time	0.200 s
Range	250 – 800 nm
Repeats	3

Table S2. Spectrometer settings for measuring excitation spectra of $K_2Gd(PO_4)(WO_4)$:20%Yb^{3+},x%Ho^{3+} phosphors.

**$K_2Gd(PO_4)(WO_4)$:20%Yb^{3+},x%Ho^{3+}**		
**Excitation scan**	**Parameters**	
	EmBW	6.00 nm
λ_{em} = **544 nm**	ExBW	1.50 nm
	Dwell	0.200 s
	Step	0.50 nm

Table S3. Spectrometer settings for measuring emission spectra of $K_2Gd(PO_4)(WO_4)$:20%Yb^{3+},x%Ho^{3+} phosphors.

**$K_2Gd(PO_4)(WO_4)$:20%Yb^{3+},x%Ho^{3+}**		
**Emission scan**	**Parameters**	
	EmBW	1.50 nm
λ_{ex} = **450 nm**	ExBW	6.00 nm
	Dwell	0.200 s
	Step	0.50 nm

Table S4. Spectrometer settings for measuring up-conversion emission spectra of $K_2Gd(PO_4)(WO_4)$:20%Yb^{3+},x%Ho^{3+} phosphors.

**$K_2Gd(PO_4)(WO_4)$:20%Yb^{3+} x% Ho^{3+}**		
**Emission scan**	**Parameters**	
	EmBW	0.25 nm
λ_{ex} **= 980 nm**	ExBW	N/A
	Dwell	0.200 s
	Step	0.50 nm

Table S5. Lattice parameters of $K_2Gd(PO_4)(WO_4)$, $K_2Gd(PO_4)(WO_4)$:10%Ho^{3+}, $K_2Gd(PO_4)(WO_4)$:20%Yb^{3+}, and $K_2Gd(PO_4)(WO_4)$:20%Yb^{3+},10%Ho^{3+} samples derived from Rietveld refinement analysis.

Sample	*a*, Å	*b*, Å	*c*, Å	*V*, Å^3	Ref.
**$K_2Ho(PO_4)(WO_4)$**	6.8820	12.1485	19.6950	1646.6	**[1]**
**$K_2Gd(PO_4)(WO_4)$**	6.94294	12.24594	19.68550	1673.7	This work
**$K_2Gd(PO_4)(WO_4)$:10%Ho^{3+}**	6.94006	12.23649	19.69193	1672.3	This work
**$K_2Gd(PO_4)(WO_4)$:20%Yb^{3+}**	6.92142	12.20985	19.67467	1662.7	This work
**$K_2Gd(PO_4)(WO_4)$:20%Yb^{3+},10%Ho^{3+}**	6.91638	12.20142	19.67897	1660.7	This work

Table S6. Effective up-conversion PL rise time and lifetime values of $K_2Gd(PO_4)(WO_4)$:20%Yb^{3+} phosphors as a function of Ho^{3+} concentration (λ_{ex} = 980 nm, λ_{em} = 660 nm).

	Rise time (µs)	Std. dev. (µs)	Lifetime τ_{eff} (µs)	Std. dev. (µs)
**Ho^{3+}(%)**	**$K_2Gd(PO_4)(WO_4)$:20%Yb^{3+},x%Ho^{3+}**			
**0.5**	29.5	1	191	2
**1**	35.1	2	161	1
**2**	38.3	3	122	1
**5**	46.4	4	105	1
**10**	59.9	7	83	3

Table S7. Effective up-conversion PL lifetime values and energy transfer efficiency (η_{tr}) of $K_2Gd(PO_4)(WO_4)$:20%Yb^{3+} phosphors as a function of Ho^{3+} concentration (λ_{ex} = 980 nm, λ_{em} = 1050 nm).

	Lifetime τ_{eff} (µs)	Std. dev. (µs)	η_{tr} (%)
**Ho^{3+}(%)**	**$K_2Gd(PO_4)(WO_4)$:20%Yb^{3+},x%Ho^{3+}**		
**0**	1279	23	-
**0.5**	435	4	65.99
**1**	406	4	68.26
**2**	322	4	74.82
**5**	306	3	76.08
**10**	278	6	78.26

Table S8. Color coordinates (CIE 1931 color space) of $K_2Gd(PO_4)(WO_4)$:20%Yb^{3+},0.5%Ho^{3+} as a function of temperature (λ_{ex} = 980 nm).

T(K)	$K_2Gd(PO_4)(WO_4)$:20%Yb^{3+},0.5%Ho^{3+}	
	CIE 1931	
	x	y
**77**	0.56516	0.43126
**100**	0.55349	0.44242
**150**	0.53167	0.46341
**200**	0.53885	0.45627
**250**	0.5563	0.43919
**300**	0.57693	0.41906
**350**	0.59753	0.39897
**400**	0.61672	0.38028
**450**	0.63227	0.36514
**500**	0.64450	0.35323

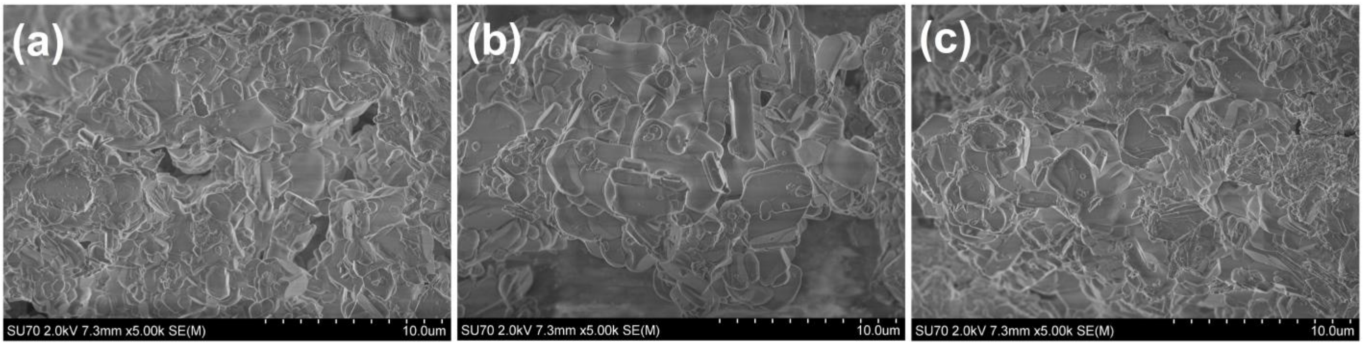

Figure S1. SEM images of $K_2Gd(PO_4)(WO_4)$:20%Yb^{3+} (a), $K_2Gd(PO_4)(WO_4)$:10%Ho^{3+} (b), and $K_2Gd(PO_4)(WO_4)$:20%Yb^{3+},10%Ho^{3+} (c).

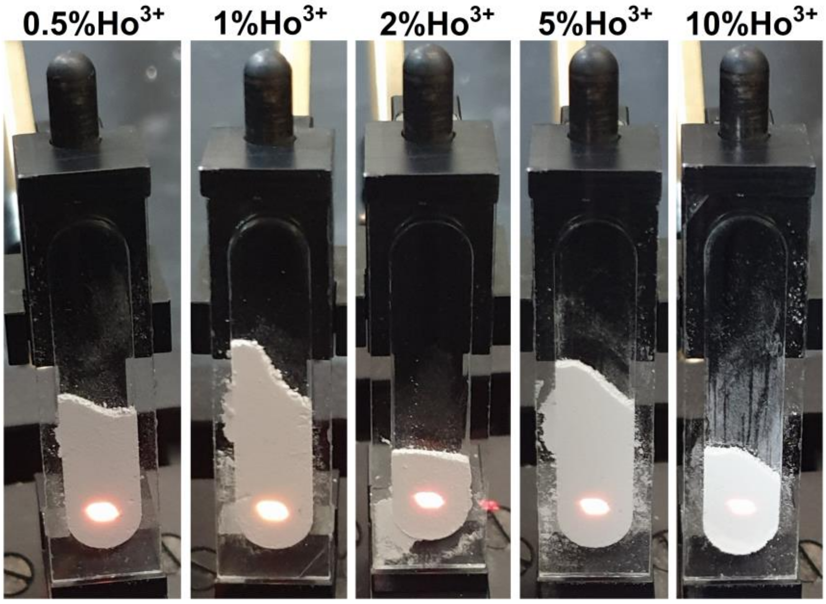

Figure S2. Digital images of the $K_2Gd(PO_4)(WO_4)$:20%Yb^{3+},Ho^{3+} up-conversion luminescence (λ_{ex} = 980 nm laser) as a function of Ho^{3+} concentration.

References

1. Terebilenko, K.V.; Zatovsky, I.V.; Baumer, V.N.; Slobodyanik, N.S.; Shishkin, O.V. $K_2Ho(PO_4)(WO_4)$. *Acta Crystallographica Section E-Crystallographic Communications* **2008**, *64*, i75, https://doi.org/10.1107/S160053680803287x.
